# Elamipretide (SS-31) improves mitochondrial dysfunction, synaptic and memory impairment induced by lipopolysaccharide in mice

**DOI:** 10.1186/s12974-019-1627-9

**Published:** 2019-11-20

**Authors:** Weixing Zhao, Zhipeng Xu, Jiangbei Cao, Qiang Fu, Yishuang Wu, Xiaoying Zhang, Yue Long, Xuan Zhang, Yitian Yang, Yunfeng Li, Weidong Mi

**Affiliations:** 1 Anesthesia and Operation Center, the First Medical Center, Chinese PLA General Hospital, 28th Fuxing Road, Haidian District, Beijing, 100853 China; 2 State Key Laboratory of Toxicology Medical Countermeasures, Beijing Key Laboratories of Neuropsychopharmacology, Beijing Institute of Pharmacology and Toxicology, Academy of Military Sciences, Beijing, 100850 China

**Keywords:** Elamipretide, SS-31, Antioxidant, Mitochondrial dysfunction, Oxidative stress, Neuroinflammation, Memory impairment, Synaptic plasticity

## Abstract

**Background:**

It is widely accepted that mitochondria have a direct impact on neuronal function and survival. Oxidative stress caused by mitochondrial abnormalities play an important role in the pathophysiology of lipopolysaccharide (LPS)-induced memory impairment. Elamipretide (SS-31) is a novel mitochondrion-targeted antioxidant. However, the impact of elamipretide on the cognitive sequelae of inflammatory and oxidative stress is unknown.

**Methods:**

We utilized MWM and contextual fear conditioning test to assess hippocampus-related learning and memory performance. Molecular biology techniques and ELISA were used to examine mitochondrial function, oxidative stress, and the inflammatory response. TUNEL and Golgi-staining was used to detect neural cell apoptosis and the density of dendritic spines in the mouse hippocampus.

**Results:**

Mice treated with LPS exhibited mitochondrial dysfunction, oxidative stress, an inflammatory response, neural cell apoptosis, and loss of dendritic spines in the hippocampus, leading to impaired hippocampus-related learning and memory performance in the MWM and contextual fear conditioning test. Treatment with elamipretide significantly ameliorated LPS-induced learning and memory impairment during behavioral tests. Notably, elamipretide not only provided protective effects against mitochondrial dysfunction and oxidative stress but also facilitated the regulation of brain-derived neurotrophic factor (BDNF) signaling, including the reversal of important synaptic-signaling proteins and increased synaptic structural complexity.

**Conclusion:**

These findings indicate that LPS-induced memory impairment can be attenuated by the mitochondrion-targeted antioxidant elamipretide. Consequently, elamipretide may have a therapeutic potential in preventing damage from the oxidative stress and neuroinflammation that contribute to perioperative neurocognitive disorders (PND), which makes mitochondria a potential target for treatment strategies for PND.

## Introduction

Postoperative cognitive dysfunction or decline, now defined as a type of perioperative neurocognitive disorder (PND), is one of the most common postoperative complications. It is characterized by a decline in cognitive function that occurs in patients after anesthesia and surgery in comparison with their preoperative cognitive status [1]. Surgery, especially in the elderly, causes central and peripheral oxidative stress and inflammatory responses that simultaneously affect synaptic plasticity, leading to cognitive dysfunction [2, 3]. However, the exact pathogenetic mechanism underlying the effect of oxidative stress and neuroinflammation on cognitive function is still unclear. Our previous studies have shown that oxidative stress and inflammatory responses play an important role in the mouse memory impairment model caused by lipopolysaccharide (LPS). Alleviating the oxidative stress and inflammatory response in the brain hippocampus can improve the learning and memory function of mice [4–6]. Notably, the free radical scavenger edaravone was found to protect against surgery and LPS administration-induced memory impairment in adult rats [7].

Mitochondria are the organelles responsible for energy metabolism and have a direct impact on neuronal function and survival [8]. Mitochondria comprise the main center for energy transfer between intracellular and extracellular compartments and play important roles in the maintenance of cell function and in apoptosis [9]. However, they are also a major source of reactive oxygen species (ROS). The activation of mitochondria-dependent apoptotic pathways and various inflammatory cytokines are mediated by mitochondrial ROS, which lead to the neuronal injury [10–12]. Oxidative-damage plays an important role in many clinical diseases such as ischemia-reperfusion injury [13, 14], neurodegenerative diseases [15–17], diabetes [18], heart failure [19], ischemic stroke [20], acute kidney injury (AKI) and chronic kidney disease (CKD) [21], age-related degenerative diseases [22], etc. Consequently, alleviating mitochondrial oxidative stress and protecting mitochondrial function provides a potential method for the treatment of these diseases.

Elamipretide (SS-31, d-Arg-Dmt-Lys-Phe-NH_2_) peptide, which was synthesized by Hazel H. Szeto and Peter W. Schiller, is a novel mitochondrial target antioxidant. Elamipretide has a dimethyltyrosine residue, allowing it to scavenge oxyradicals and inhibit linoleic acid and low-density lipoprotein oxidation [23]. This mitochondrial antioxidant peptide has the ability to eliminate ROS and increase adenosine triphosphate (ATP) in mitochondria, thus maintaining the mitochondrial membrane potential (MMP). By reducing mitochondrial ROS, elamipretide is able to prevent opening of the mitochondrial permeability transition pore (mPTP), prevent mitochondrial swelling, and reduce cytochrome c release in response to a high Ca^2+^ overload [24, 25]. Elamipretide is currently used as a strategy to protect against neurodegenerative diseases, inflammatory diseases, and ischemia-reperfusion injury in animal research [16, 17, 19, 26–29].

Lipopolysaccharide (LPS, endotoxin), a cell wall component of Gram-negative bacteria, is a major bacterial toll-like receptor 4 (TLR4) ligand that activates the innate immune response to infections. It has been shown to induce hippocampal oxidative stress, a neuroinflammatory response, neuronal death via apoptosis, and ultimately memory impairment, and has been widely used in preclinical models to investigate postoperative cognitive dysfunction [4, 6, 30].

Based on the above findings, we hypothesized that during neuroinflammation, elamipretide might protect against mitochondrial dysfunction, attenuate oxidative stress and the inflammatory response in the hippocampus, and thus improve memory impairment. To test this hypothesis, we assessed the neuroprotective effects of elamipretide against LPS-induced oxidative damage and neuroinflammation in the present study. The results obtained may provide the basis for new treatment strategies for PND by making mitochondria a potential target of treatment.

## Materials and methods

### Animals

Adult male C57BL/6 mice aged 10–11 weeks and weighing 21–23 g were purchased from Vital River Laboratories Animal Technology Co. Ltd. (Beijing, China. Permit Number: SCXK (JING) 2012-0001). Mice were housed in groups of three to five per plastic cage (24 × 36 × 24 cm) under standard environmental conditions (temperature 24 ± 1 °C, a 12 h light-dark cycle, and 50% ± 10% humidity) with ad libitum access to food and water. All experimental procedures and protocols were reviewed and approved by the Institutional Animal Care and Utilization Committee (IACUC) of the Chinese PLA General Hospital (Beijing, China) and were performed in accordance with the Guidelines for the Care and Use of Laboratory Animals of the National Institutes of Health, USA. The animals were acclimatized for 7 days before the experiment and were group-housed with the same cage mates throughout the acclimation and testing periods, and adequate measures were taken to minimize animal discomfort.

### Experimental protocols

Ninety-six mice were randomly assigned to one of the following four treatment groups (*n* = 24 mice per group): a control plus placebo group (CON group), a control plus elamipretide group (SS-31 group), LPS plus placebo group (LPS group), and the LPS plus elamipretide group (S + L group).

LPS (Sigma, St. Louis, MO, USA) was dissolved in artificial cerebrospinal fluid (aCSF: 140 mM NaCl; 3.0 mM KCl; 2.5 mM CaCl_2_; 1.0 mM MgCl_2_; and 1.2 mM Na_2_HPO_4_; pH = 7.4) (2 μg in 2 μL), or 2 μL aCSF was stereotaxically infused into the lateral ventricle at the coordinate: AP − 0.5, ML + 1.0, and DV − 2.0, using a mouse brain stereotactic apparatus (Kopf Instruments, Tujunga, CA, USA) after anesthetizing the mice with ketamine/xylazine (200/10 mg/kg). Using a 10 μL Hamilton microsyringe, the injection speed was set at 0.667 μL/min, and the needle was held in place for 2 min following injection for proper dispersal of the drug from the tip. Mice were treated intraperitoneally with phosphate-buffered saline or elamipretide (5 mg/kg, synthesized in China by Peptides Co., Ltd., Shanghai) 30 min before the stereotaxic injection, and then once daily for three consecutive days thereafter. Injections (0.2 mL/10 g weight; 0.025% concentration) were alternated daily between the right and left side of the abdomen. The dose of elamipretide was chosen based on previous research showing that repeated injections of elamipretide 5 mg/kg provided maximum neuroprotective effects without any adverse effects in mouse models [26, 31].

### Behavior tests

All assays were conducted during light-phase hours (9:00–17:00) by observers who did not know the group of the mice until the test had been completed.

#### Open field test

The open field tests were performed 2 h before the probe tests for reference memory, according to a previous study [32]. The mice were individually placed in the apparatus consisting of four transparent Plexiglas arenas (50 × 50 × 30 cm) in which the floor and walls were covered with white paper to prevent any interference from movements of the neighboring mouse. Each mouse was released into a corner of the box and allowed to explore for 60 min. The total grid crossing and velocity, duration, and distance in the center area were recorded during first 10 min, which were analyzed using the ANY-maze behavioral tracking software system (Stoelting Co. Wood Dale, IL, USA). The open field apparatus was thoroughly cleaned with 5% ethyl alcohol after the test of each mouse and allowed to dry between tests.

#### Morris water maze

The Morris water maze (MWM) test, which is a hippocampal-dependent test of spatial learning, spatial memory, and cognitive flexibility for rodents, was performed as described previously with minor modifications [6, 32]. The water maze was a white circular tank made of polyvinyl chloride (118 cm in diameter and 42 cm in height) and filled with white non-toxic paint and water (23 ± 1 °C) to a depth of 28 cm. The maze was placed in a room with several visual cues for orientation in the maze. The maze was divided into four quadrants, i.e., the first, second, third, and fourth quadrants. An invisible platform (11 cm in diameter) was placed 1 cm below the water surface in the first quadrant (target quadrant). Mice were trained for the hidden platform version during acquisition, which consisted of four trials for five consecutive days. Mice were released to the MWM from all the other three quadrants, except the target quadrant, and were trained to find the hidden platform and climb onto it within 60 s. When the mice failed to reach the platform within 60 s, they were manually guided to the platform and allowed to stand on the platform for 15 s before being transferred back to cages. After that, the mouse was removed to its cage and the second animal was tested. This rotation was repeated until all animals had completed a trial. This process was repeated for subsequent trials until four trials had been completed per day for five consecutive days. After each trial, animals were towel dried and returned to their home cage under a heater for 10 min.

The probe trial was performed on the next day after stereotactic microinjection (day 7) in the water maze without a platform. Swimming velocity, platform-site crossings, latency to the platform, and the percentage of time traveled in the different quadrants were recorded in a single 60-s trial. Reference memory was determined by a preference for the platform area. The trajectories of the mice were recorded and analyzed using ANY-maze behavioral tracking software system (Stoelting Co. Wood Dale, IL, USA).

On days 7, 8, and 9, working memory was tested, during which both the platform and mice were randomly placed in a novel position to assess working- or trial-dependent learning and memory. In this procedure, the animal is given two trials per day. On each day, animals underwent the first training trial to ensure that all mice learned the new platform location. After 15 s, the second trial as the test trial or matching trial was performed, in which each mouse was released from the same location as in the first trial; if it recalled the first trial, the mouse would swim a shorter path to the platform in the second trial. As the platform is moved daily, learning of platform position from the previous day cannot be transferred to the next day; hence, recall on each day during the second trial is dependent on that day’s training trial and measures only temporary or working memory. Measurement of data in the second trial was conducted up to 60 s, and the latency for mice that could not reach the platform in the allotted time was regarded as 60 s. Eventually, the escape latency to the platform in the second trial was recorded as measure of temporary or working memory. The trajectories of the mice were recorded and analyzed using ANY-maze behavioral tracking software system (Stoelting Co. Wood Dale, IL, USA).

#### Contextual fear conditioning test

To measure the ability of hippocampus-dependent learning and memory, we employed the fear conditioning paradigm (30 cm long × 26 cm wide × 22 cm high; XR-XC404, Softmaze Information Technology Co. Ltd., Shanghai, China). The contextual fear conditioning test was performed as described previously with minor modifications [32]. On the acquisition day, mice were habituated for 2 min, received a 2-s 0.5 mA foot shock every 2 min four times, and were then returned to cages 2 min after the fourth shock. The day 2 trial was performed on the next day after stereotactic microinjection in the same chamber for 6 min to evaluate consolidated fear memory by analyzing the time of freezing.

### Tissue isolation

As previously described by us [4, 6], at 6 h after injection of LPS, six mice in each group were decapitated. The hippocampus was rapidly removed for an enzyme-linked immunosorbent assay (ELISA). The brains of six mice sacrificed at 24 h in each group were quickly removed and washed in ice-cold saline. The hippocampus was dissected and separated into two halves for mitochondria isolation and immunoblotting analysis. Tissue for immunoblotting analysis was collected carefully in a sterile tube prior to being snap-frozen in liquid nitrogen and stored at − 80 °C until analysis. Transcardial perfusion was performed with ice-cold standard phosphate-buffered saline (PBS) in another six mice at 24 h and 72 h within each group. Brains were fixed in 4% paraformaldehyde for 48 h thereafter for terminal transferase biotinylated-dUTP nick end labeling (TUNEL) and Golgi-staining analysis, respectively. All mice were sacrificed by cervical decapitation under deep isoflurane anesthesia.

### Isolation of hippocampal mitochondria

Mitochondria from the aforementioned half of the hippocampus were isolated using the tissue mitochondria isolation kit for tissue protocols (C3606, Beyotime Institute of Biotechnology, Shanghai, China). Briefly, fresh tissue samples were homogenized in ice-chilled Dounce homogenizers (1:10, w/v) using an isolation buffer and centrifuged at 1000 g for 5 min at 4 °C. Supernatants were then removed and centrifuged at 3500 g for 10 min at 4 °C. The resulting sediment consisted of mitochondria. The supernatants were collected and centrifuged at 12,000 g for 10 min at 4 °C to remove sediment and to obtain cytoplasmic proteins. Appropriate amounts of mitochondrial stock solution were added to resuspend the sediment, and the protein of the mitochondria was determined by the Micro BCA protein assay kit (Beyotime Institute of Biotechnology, Shanghai, China).

### Determination of the mitochondrial membrane potential level

A mitochondrial membrane potential (MMP) assay kit with 5,5′,6,6′-tetrachloro-1,1′,3,3′ tetraethylbenzimidazolyl-carbocyanine iodide (JC-1) [C2006; Beyotime Institute of Biotechnology, Shanghai, China] was used to detect MMP. JC-1 accumulates to form J-aggregates and emits red fluorescence (Cy3, excitation/emission wave length of 525/590 nm) in mitochondria with higher membrane potentials, while JC-1 monomers emit green fluorescence (fluorescein isothiocyanate [FITC], excitation/emission wave length of 490/530 nm) in mitochondria with lower membrane potentials. A decrease of the ratio (red: green) was thus interpreted as a decrease in MMP [33]. For this assay, different groups of 0.1 mL of purified mitochondria (protein concentration 0.2 mg/mL) were incubated with 0.9 mL of 0.2X JC-1 staining working solution. A time scan was performed directly using a fluorescence microplate reader (Gemini EM Microplate Reader, Molecular Devices, Sunnyvale, CA, USA) with an excitation/emission wave length of 485/590 nm, and observed under an Olympus BX5 fluorescence microscope imaging system (Olympus America, Melville, NY, USA).

### Adenosine triphosphate assay

Adenosine triphosphate (ATP) content was measured by a firefly luciferase-based ATP assay kit (S0026, Beyotime Institute of Biotechnology, Shanghai, China) with some modifications [34]. According to the manufacturer’s instructions, cold lysis buffer (20 mg/200 μL) was quickly added to freshly harvested hippocampal tissue. Tissues were homogenized with a glass homogenizer on ice and centrifuged at 12,000 g for 5 min at 4 °C. The detection wells had 100 μL of ATP testing working solution added, were incubated for 5 min at room temperature, and then added to a 20 μL sample or standard. The protein concentration was measured with BCA protein assay kit (Beyotime Institute of Biotechnology, Shanghai, China). Emitted light was linearly related to the ATP concentration and was measured using a microplate luminometer (GloMax® 96, Promega Corporation, WI 53711, USA). Measurements were conducted in duplicate. The mean value of the duplicates was used to represent the value of each mouse. Data were normalized to the control group and expressed as a percentage of control levels.

### Assessment of reactive oxygen species

The GENMED oxidative stress reactive oxygen species (ROS) primary fluorescence assay kit for living tissue (GMS10016.4, GENMED Scientifics Inc., MA, USA) was chosen to detect ROS. 2′,7′-Dichlorofluorescein diacetate (DCFH-DA) is a stain that can penetrate through the cell membrane freely. Green fluorescence is produced once DCFH-DA is oxidized by hydrogen peroxide, peroxide groups, peroxynitrite, etc. Briefly, the hippocampus was freshly harvested 24 h after stereotaxic injection and cold GENMED diluent (10 mg/100 μL) was quickly added. Tissues was homogenized with a DOUNCE homogenizer on ice. The protein in the hippocampus was determined by the GENMED Bradford protein assay kit (GMS30030.1, GENMED Scientifics Inc., MA, USA). Fifty microliters of the supernatant was mixed with 50 μL catalyst in a well of 96-well plates and incubated at room temperature for 5 min. Subsequently, GENMED staining working solution containing DCFH-DA (100 μL) was added to each well and reacted for 20 min in the dark. Fluorescence at 490 nm excitation and 520 nm emission was read on a fluorescence microplate reader (Gemini EM Microplate Reader, Molecular Devices, Sunnyvale, CA, USA) and observed under an Olympus BX5 fluorescence microscope imaging system (Olympus America, Melville, NY, USA).

### Biochemical analysis

#### Malondialdehyde

Malondialdehyde (MDA) is a degraded oxidative lipid product from cell membranes and is used as a reliable indicator of oxidative stress [35]. The amount of MDA was measured by the reaction of one molecule of MDA with two molecules of thiobarbituric acid (TBA) to yield a pink colored chromogen. The color reaction was measured at 532 nm with a reference wave length of 450 nm. The levels of MDA in the hippocampi of mice were measured using commercial assay kits (Beyotime Biotechnology Institute, Nantong, China) according to the manufacturer’s instructions.

#### Superoxide dismutase activity

Superoxide dismutase (SOD) is an endogenous scavenger of reactive oxygen species (ROS) and is one of the major antioxidant enzymes involved in protecting the nerve tissue from oxidative stress. The activity of SOD was measured by the reaction of NBT (nitro blue tetrazolium) with two molecules of superoxide anion to yield a blue colored chromogen. SOD has the ability to inhibit the superoxide anion-free radical O_2_^−^. The color reaction was measured at 560 nm with a reference wave length of 650 nm. The SOD activity of tissue was also measured using commercial assay kits (Beyotime Biotechnology Institute, Nantong, China) according to the manufacturer’s instructions.

### ELISA

Concentrations of interleukin-6 (IL-6) and tumor necrosis factor α (TNF-α) were examined using an ELISA kit (NEOBIOSCIENCE, Beijing, China). Hippocampal tissue was homogenized in a mixture of phenylmethylsulfonyl fluoride (PMSF) and radioimmunoprecipitation assay (RIPA) lysis buffer (Solarbio Science & Technology Co., Ltd., Beijing, China)—10 μL PMSF: 1 mL RIPA lysis buffer on ice. Supernatant protein concentrations were determined after centrifugation at 12,000 rpm for 5 min at 4 °C with a BCA Protein Assay kit (Beyotime Institute of Biotechnology, Shanghai, China). For each sample, 5 μL of extracted protein was used for detection. The procedure followed the manufacturer’s instructions. The absorbance was read on a spectrophotometer at a wave length of 450 nm. The concentrations of IL-6 and TNF-α were calculated according to the standard curve and recorded as pg/mg protein.

### Western blotting

Hippocampal tissue was homogenized in a mixture of PMSF and RIPA lysis buffer (Solarbio Science & Technology Co., Ltd., Beijing, China)—10 μL PMSF: 1 mL RIPA lysis buffer on ice. Supernatant protein concentrations were determined after centrifugation at 12,000 rpm for 5 min at 4 °C with a BCA Protein Assay kit (Beyotime Institute of Biotechnology, Shanghai, China). Proteins, 20 μg per lane (specified in figure legends), were separated on gradient sodium dodecyl sulfate–polyacrylamide gels (SDS–PAGE) and transferred onto a polyvinylidenedifluoride (PVDF) membrane (Millipore). Blots were blocked in 5% non-fat milk or bovine serum albumin (BSA) for 2 h and probed with primary antibodies overnight at 4 °C. The primary antibodies used in this study were rabbit anti-BDNF IgG (1:1000, catalog number: ab108319, Abcam), rabbit anti-phospho-TrkB IgG (Tyr816) (1:500, catalog number: # ABN1381, Millipore), rabbit anti-TrkB IgG (1:1000, catalog number: # 4603, Cell Signaling Technology), rabbit anti-postsynaptic density protein 95 (PSD-95) antibody (1:1000, catalog number: ab18258, Abcam), rabbit anti-synaptophysin (SYN) IgG (1:20000, catalog number: ab32127, Abcam), and rabbit anti-GAPDH IgG (1:10000, catalog number: ab181602, Abcam). After washing 5× in TBS-tween, horseradish peroxidase (HRP)-conjugated secondary antibodies (Cell Signaling Technology) were applied for 1 h, rinsed again, and bands detected using ECL (Santa Cruz or Millipore). The images were digitized from the membrane and the band intensity was quantified using Gel-Pro Analyzer software, Version 3.1 or ImageJ software, version 2.0.0.

### Terminal Transferase Biotinylated-dUTP Nick end labeling

The terminal transferase biotinylated-dUTP nick end labeling (TUNEL) method was performed to label cells undergoing apoptosis following the manufacturer’s instructions (Roche Applied Science, Penzberg, Germany). Briefly, the brain sections were incubated in a permeabilization solution and then incubated with a TUNEL reaction mixture. Finally, the sections were incubated with 10 μg/mL Hoechst 33342 in a humidified dark chamber. After the TUNEL method, the sections were stained with DAPI (4,6-diamidino-2-phenylindole) and mounted with Fluoromount (SouthernBiotech, Birmingham, AL, USA), and FITC-labeled apoptotic cells were then imaged on an Olympus BX5 fluorescence microscope imaging system (Olympus America, Melville, NY, USA). The number of apoptotic neural cells per view was counted using microscopy at × 400 magnification.

### Golgi–Cox staining

Golgi–Cox staining was used to detect the dendritic spines of hippocampal neurons as described previously, with modification [36]. The half brain was dissected out and processed with Golgi–Cox Impregnation & Staining System according to the manufacturer’s instruction (super Golgi Kit, Bioenno Tech, LLC). After impregnation, sections (100 to 200 μm) were obtained using a vibratome, and the sections were mounted on gelatin-coated glass slides and stained. Images were taken by using a Zeiss Imager II deconvolution microscope with SlideBook 5.5 software. For quantification of spines, images were acquired as a series of z-stack at 0.1 μm steps to create sequential images, enabling spine counting and spine morphology measurements on 3D images using a × 100 oil objective. ImageJ (Version 2.0.0-rc-69/1.52n https://imagej.nih.gov/ij/index.html) and NeuronStudio (Version 0.9.92, http://research.mssm.edu/cnic/tools-ns.html) were used to reconstruct and analyze dendritic spines, as described previously [37]. Spine densities were estimated by counting the number of spines along 100–150 μm (CA1) and 50–75 μm (DG) segments of dendrites in hippocampal neurons. Three neuronal cells per brain slice, and three brain slices per animal were chosen for quantitative analysis. The number of spines was counted by double-blind hand counter.

### Statistical analysis

All data were analyzed by an observer who was blind to the experimental protocol. Statistical calculations were performed using the statistical analysis software GraphPad Prism, Version 7.0 (GraphPad, San Diego, CA, USA). Data were expressed as the mean ± standard error (SE). Intergroup comparisons were conducted by two-way analysis of variance (ANOVA) [LPS × elemipretide] followed by Tukey’s post hoc test to determine significant differences between experimental groups. For acquisition training (days 1 to 5) of the MWM, data were analyzed using a two-way ANOVA (treatment × trial time) with repeated measures (trial days) followed by a Bonferroni post hoc test to analyze the difference in escape latency between each group. *P* values < 0.05 were considered statistically significant.

## Results

### General behavioral performances and anxiety responses were not changed by LPS and elamipretide treatment

**Fig. 1:**
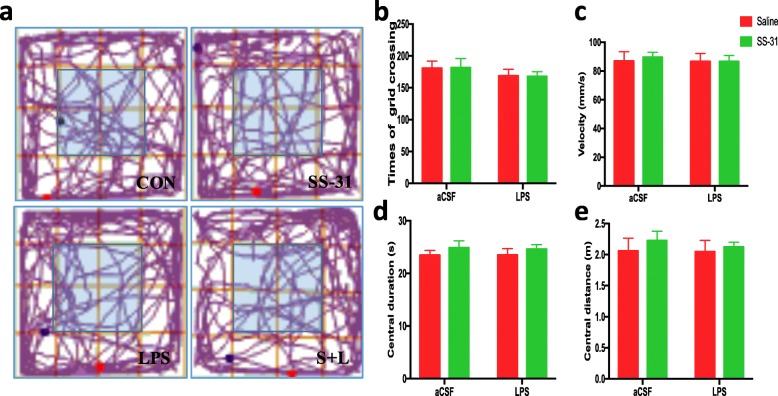
Determining the effects of elamipretide (SS-31) and LPS on mice in the open field test. **a** Representative moving traces in the open arena during the first 10 min. Times of grid crossings **b**, velocity **c** in the whole arena, and duration of stay **d**, distance of movement **e** in the center (light blue region) are expressed as the mean ± SE (*n* = 10). *SS*-*31* elamipretide, *aCSF* artificial cerebrospinal fluid, *LPS* lipopolysaccharide, *SE* standard error

Before the MWM task and contextual fear conditioning test, general behavioral performances and anxiety responses were assessed by the open field test. As shown in Fig. 1, times of grid crossings (b), velocity (c) in the whole arena, duration of stay (d), and distance of movement (e) in the center (light blue region) were recorded. Spontaneous locomotor activity, as determined by times of grid crossings and velocity in the whole arena, and exploratory behaviors in the center zone (as determined by the duration of stay and distance of movement) were comparable among all the experimental groups. No significant difference was observed in general behavioral performances and the anxiety responses after LPS and elamipretide treatment between the animals in the CON, elamipretide, LPS, and S + L groups (Fig. 1b), suggesting that the impaired performance in the LPS group was not a result of reduced locomotor ability.

### Elamipretide prevented hippocampus-dependent learning and memory impairment induced by LPS

**Fig. 2:**
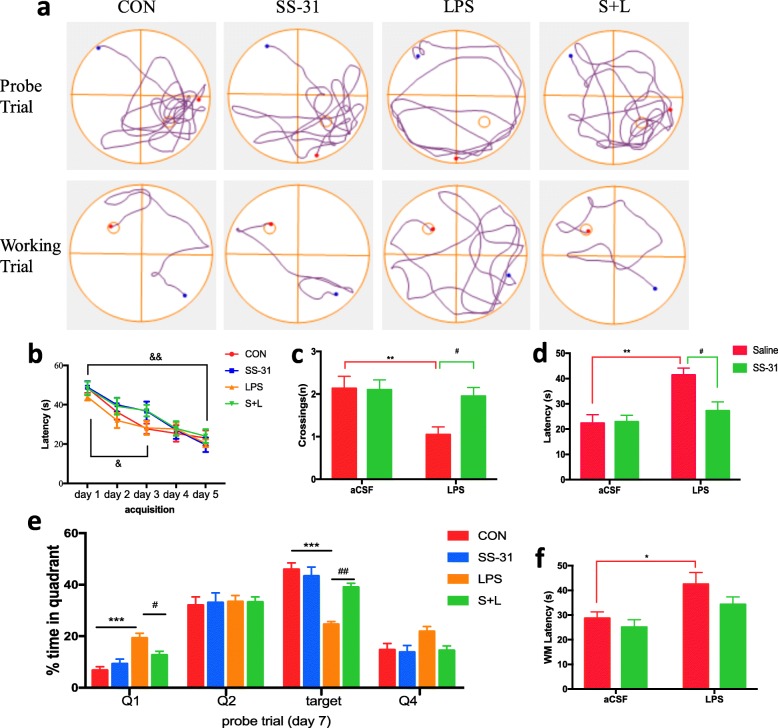
Elamipretide (SS-31) improved behavioral performance in MWM after exposure to LPS in mice. **a** Representative trajectories of mice from each experimental group in the probe trial and working trial. During the probe trial, the hidden platform was removed, and during the spatial working memory trial, the hidden platform was changed to the center of the opposite quadrant. The circle represents the previous location of the platform (Probe trial) and the new place of the hidden platform (Working trial). **b** Each experimental group of mice learned to locate the hidden platform by using the surrounding cues after 5 days of training. In the probe trial, platform-site crossings **c**, latency to arrive at the primary platform-site **d**, and the percentage of time that the mouse spent navigating each quadrant **e** were analyzed. **f** Latency to get on the new platform during the spatial working memory trial was measured in the indicated group of mice. Data are expressed as the mean ± SE (CON/LPS, 10/15 mice). *P < 0.05, **P < 0.01, ***P < 0.001 vs. CON group; #*P* < 0.05, ##*P* < 0.01 vs. LPS group; &*P* < 0.05, &&*P* < 0.01 vs. acquisition day 1. *SS*-*31* elamipretide, *aCSF* artificial cerebrospinal fluid, *LPS* lipopolysaccharide, *SE* standard error

Our previous work has demonstrated that intracerebroventricular administration of LPS leads to learning and memory deficits [4, 6]. Therefore, the protective effects of elamipretide on LPS-induced memory deficits were examined in this model. For the MWM test, all four experimental groups of mice learned to locate a hidden platform by using visual cues around the maze, as evidenced by the decreasing latencies over the training period (*F*_(4, 218)_ = 29.6, *P* < 0.001); but no difference was observed between the groups during the same day, indicating that the groups did not differ in spatial learning (Fig. 2b). On day 7 of the probe trial, the platform was removed and platform-site crossings (Fig. 2c), latency to arrive at the primary platform-site (Fig. 2d), and the amount of time the mice spent in the target quadrant where the platform was previously placed (Fig. 2e) were recorded. It was observed that elamipretide prevented spatial memory deficits caused by LPS (*F*_LPS_ = 7.53, *F*_SS-31_ = 3.7, *F*_LPS × SS-31_ = 4.31 for platform-site crossings, *P* < 0.05; *F*_LPS_ = 12.8, *F*_SS-31_ = 4.3, *F*_LPS × SS-31_ = 5.03 for latency to arrive at primary platform-site, *P* < 0.05; *F*_LPS_ = 37.8, *F*_SS-31_ = 8.1, *F*_LPS × SS-31_ = 16.7 and for percentage of time traveled in the target quadrant during the probe trial, *P* < 0.05; Fig. 2c, d, e).

In the spatial working memory trial, both the platform and mice were randomly placed in a novel position to assess working- or trial-dependent learning and memory. If the animal recalls the sample trial, it will swim a shorter path to the goal on the second trial. It was observed that the escape latency needed to reach the new platform was significantly increased in the LPS group (*F*_LPS_ = 11, *P* = 0.0375; Fig. 2f) compared with the CON group. In contrast, the escape latency was returned to the CON group levels in mice treated with elamipretide.

**Fig. 3:**
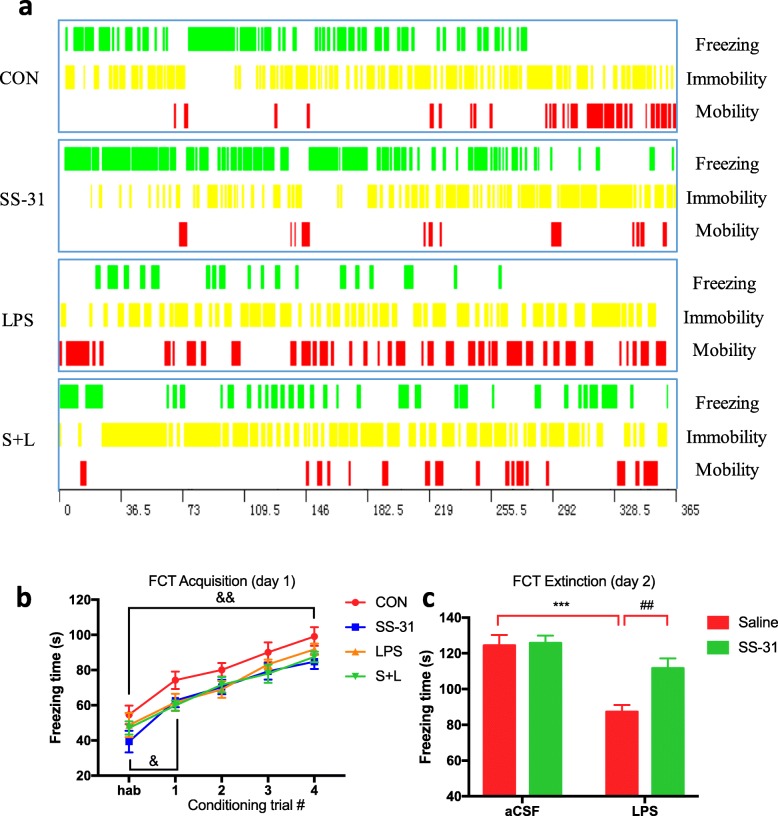
Elamipretide (SS-31) increased the consolidated long-term fear memory in LPS-treated mice. **a** Representative time distribution of freezing, immobility and mobility on day 2 of the fear conditioning (FCT) extinction test in indicated groups of mice. **b** In the contextual fear conditioning, the freezing response during habituation (hab) and acquisition were analyzed for 2 min per trial. A foot shock was given at the end of habituation and the first three conditioning trials. **c** The freezing time was measured in the chamber for 6 min without reinforcing the shock on day 2 of the FCT extinction test. Data are expressed as the mean ± SE (*n* = 6–8). ***P < 0.001 vs. CON group; ##*P* < 0.01 vs. LPS group; &*P* < 0.05, &&*P* < 0.01 vs. hab. *FCT* fear conditioning test, *SS*-*31* elamipretide, *aCSF* artificial cerebrospinal fluid, *LPS* lipopolysaccharide, *SE* standard error

In the contextual fear conditioning test, mice learned to express a fear response (i.e., freezing) after linking the chamber environment (CS, conditioned stimulus) with a harmful electric shock (US, unconditioned stimulus), a process called acquisition. The freezing levels among the experimental groups were similar during habituation, and increased after CS-US paired trainings (i.e., acquisition, *F*_(4, 104)_ = 114, *P* < 0.001), so all groups of mice were able to learn associated tasks (Fig. 3b). However, the consolidated long-term fear memory, determined on the next day without US, was significantly reduced in LPS group (*F*_LPS_ = 26.1, *P* < 0.001; Fig. 3c) compared with the CON group, while elamipretide significantly increased the fear memory (*F*_SS-31_ = 6.63, *P* = 0.0093; Fig. 3c) in LPS-treated mice.

Collectively, our results suggest that LPS causes hippocampus-dependent learning and memory impairment, which can be ameliorated by elamipretide treatment.

### Elamipretide protected the hippocampus against LPS-induced mitochondrial dysfunction by maintaining MMP and ATP levels

**Fig. 4:**
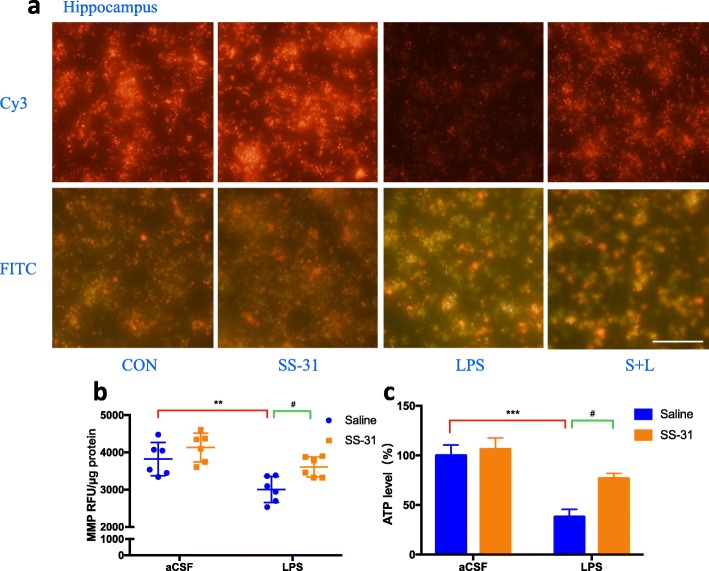
Protective effects of elamipretide (SS-31) on mitochondrial dysfunction induced by LPS in the mouse hippocampus. **a** Representative fluorescence intensity images of J-aggregates (Cy3) and JC-1 monomers (FITC) from hippocampal mitochondria in each group. The MMP level **b** and ATP production **c** in the hippocampus were measured immediately after samples were prepared from each group. Scale bars: 20 μm, Data are expressed as the mean ± SE (*n* = 6). **P < 0.01, ***P < 0.001 vs. CON group. #*P* < 0.05 vs. LPS group. *SS*-*31* elamipretide, *aCSF* artificial cerebrospinal fluid, *LPS* lipopolysaccharide, *SE* standard error

We examined the effects of elamipretide on MMP and ATP levels. Our results showed that the fluorescence intensity under Cy3 (detect J-aggregates) in the LPS group was much weaker than in the CON group, which means lower membrane potentials, but elamipretide significantly increased the fluorescence intensity in the S + L group (Fig. 4a). Consistently, the fluorescence intensity under FITC (detect JC-1 monomers) in the LPS group was much higher than in the CON group, which also means lower membrane potentials, but elamipretide significantly decreased the fluorescence intensity in the S + L group (Fig. 4a). The results of the fluorescence microplate reader and microplate luminometer indicated that the level of MMP (*F*_LPS_ = 19.6, *P* = 0.005; Fig. 4b) and ATP (*F*_LPS_ = 25.8, *P* < 0.001; Fig. 4c) were decreased in the LPS group when compared with the CON group. Conversely, elamipretide reversed the effects on MMP (*F*_SS-31_ = 9.24, *P* = 0.04; Fig. 4b) and ATP levels induced by LPS (*F*_SS-31_ = 6.41, *P* = 0.03; Fig. 4c), indicating that elamipretide timely reversed LPS-induced mitochondrial dysfunction.

### Elamipretide attenuated oxidative stress and the inflammatory response induced by LPS in the mouse hippocampus

**Fig. 5:**
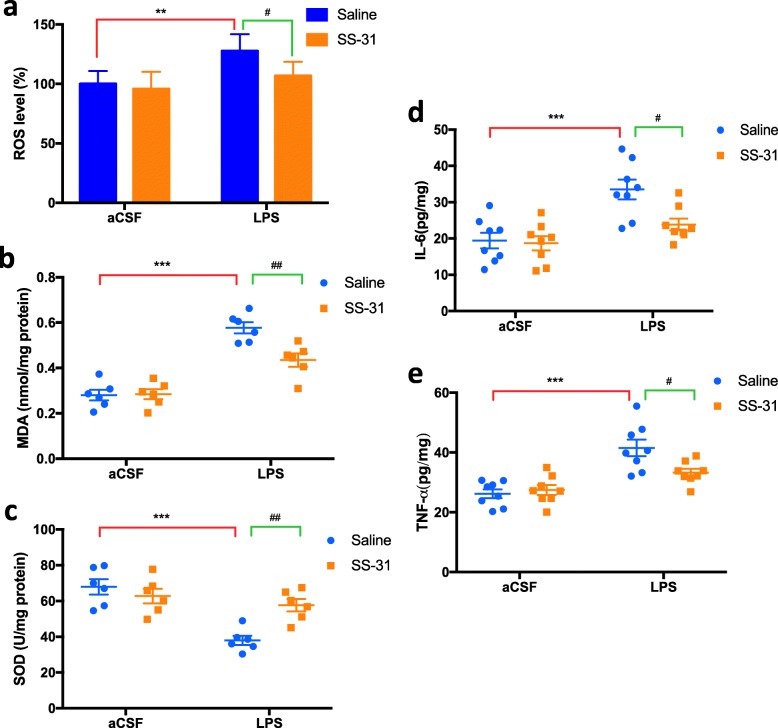
Elamipretide (SS-31) attenuated oxidative stress and the inflammatory response induced by LPS in the mouse hippocampus. **a** Reactive oxygen species (ROS) levels, **b** malondialdehyde (MDA) levels, and **c** superoxide dismutase (SOD) activities. ELISA assays of IL-6 **d** and TNF-α **e** levels in samples of the hippocampus. Scale bars: 20 μm, Data are expressed as the mean ± SE (*n* = 6–8). **P < 0.01, ***P < 0.001 vs. CON group; #*P* < 0.05, ##*P* < 0.01 vs. LPS group. *SS*-*31* elamipretide, *aCSF* artificial cerebrospinal fluid, *LPS* lipopolysaccharide, *SE* standard error

Considering that mitochondrial dysfunction might result in oxidative stress and an inflammatory response in the LPS-treated hippocampus, we further determined the effects of elamipretide on hippocampal ROS, MDA levels, SOD activities, and two important pro-inflammatory cytokines, TNF-α and IL-6. The fluorescence intensity of oxidized dichloro-dihydrofluorescein diacetate (DCFH-DA) in the LPS group was much higher than in the CON group, which indicates increased ROS, but elamipretide significantly decreased the fluorescence intensity in the S + L group. Compared with the CON group, LPS induced a significant increase in the levels of ROS (*F*_LPS_ = 13.6, *P* = 0.007; Fig. 5a), MDA (*F*_LPS_ = 79.6, *P* < 0.001; Fig. 5b), IL-6 (*F*_LPS_ = 20.1, *P* < 0.001; Fig. 5d), and TNF-α (*F*_LPS_ = 31.3, *P* < 0.001; Fig. 5e). Interestingly, elamipretide significantly attenuated abnormally increased levels of ROS (*F*_SS-31_ = 5.74, *P* = 0.05; Fig. 5a), MDA (*F*_SS-31_ = 7.63, *P* = 0.003; Fig. 5b), IL-6 (*F*_SS-31_ = 5.83, *P* = 0.018; Fig. 5d), and TNF-α (*F*_SS-31_ = 3.42; *P* = 0.022, Fig. 5e) in the hippocampus of the S + L group. As demonstrated in Fig. 5c, LPS induced a significant decrease in SOD activity (*F*_LPS_ = 23, *P* < 0.001) in the hippocampus compared with the CON group; this abnormal decrease in SOD activity was largely prevented by elamipretide (*F*_SS-31_ = 3.92, *P* = 0.006).

Our results suggested that elamipretide attenuated oxidative stress and the inflammatory response induced by LPS in the mouse hippocampus.

### Elamipretide significantly decreased neural cell apoptosis in the hippocampus of LPS-treated mice

**Fig. 6:**
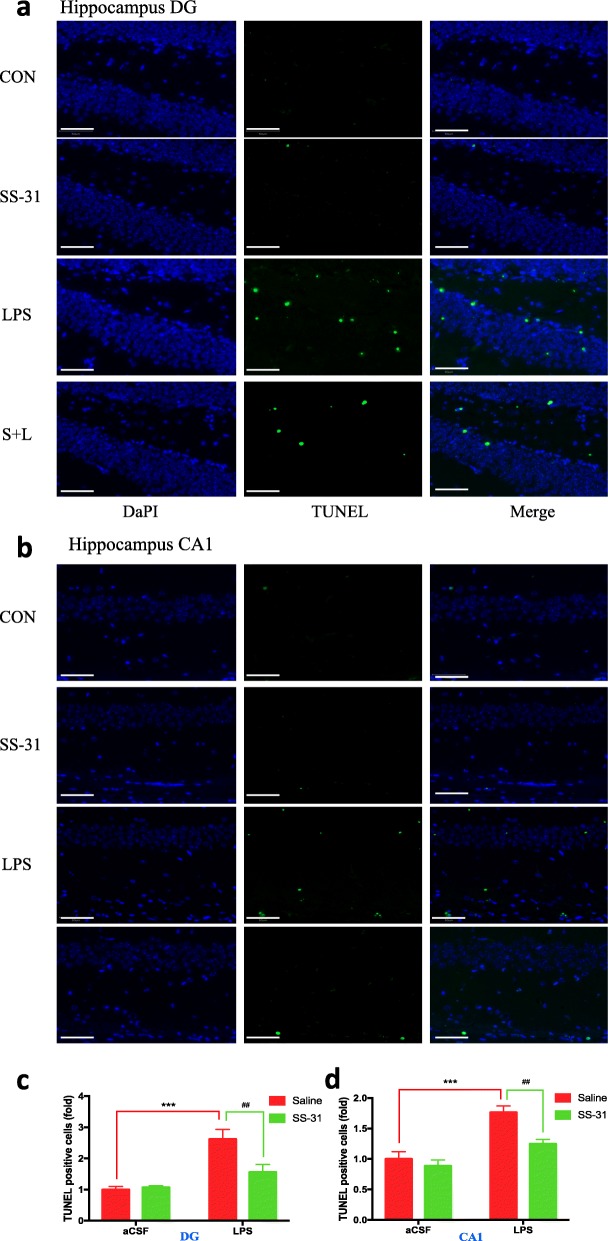
Effect of elamipretide (SS-31) on neural cell apoptosis in mice with hippocampal impairment. **a**, **b** TUNEL staining demonstrated neural cell apoptosis in the DG and CA1 regions of the hippocampus from indicated group of mice. **c**, **d** The number of hippocampal apoptotic neural cells in the DG and CA1 regions of the hippocampus from indicated group of mice. Scale bars: 50 μm. Data are expressed as the mean ± SE (*n* = 6). ***P < 0.001 vs. CON group; ##*P* < 0.01 vs. LPS group. *SS*-*31* elamipretide, *aCSF* artificial cerebrospinal fluid, *LPS* lipopolysaccharide, *SE* standard error

In order to observe the effect of elamipretide on LPS-induced hippocampal neural cells, we carried out a TUNEL assay. We quantified the TUNEL-positive cells using fold change. One-fold refers to the ratio of TUNEL-positive cells to the total cells in CON group. We found that LPS intraventricular injection increased TUNEL-positive cells (apoptosis) as compared with the CON group in brain hippocampal tissues of the DG region (2.62-fold versus 1-fold, *P* < 0.001; Fig. 6a, c) and CA1 region (1.77-fold versus 1-fold; *P* < 0.001; Fig. 6b, d). Consistent with the above findings, elamipretide significantly attenuated LPS-induced neural cell apoptosis in the hippocampal DG region (1.56-fold versus 2.62-fold, *P* < 0.01; Fig. 6a, c) and the CA1 region (1.25-fold versus 1.77-fold, *P* = 0.01; Fig. 6b, d).

These results suggested that elamipretide significantly reduced neural cell apoptosis in the hippocampus of LPS-treated mice.

### Elamipretide enhanced the hippocampal BDNF pathway and synaptic structural complexity in mice with LPS-induced hippocampal impairment

**Fig. 7:**
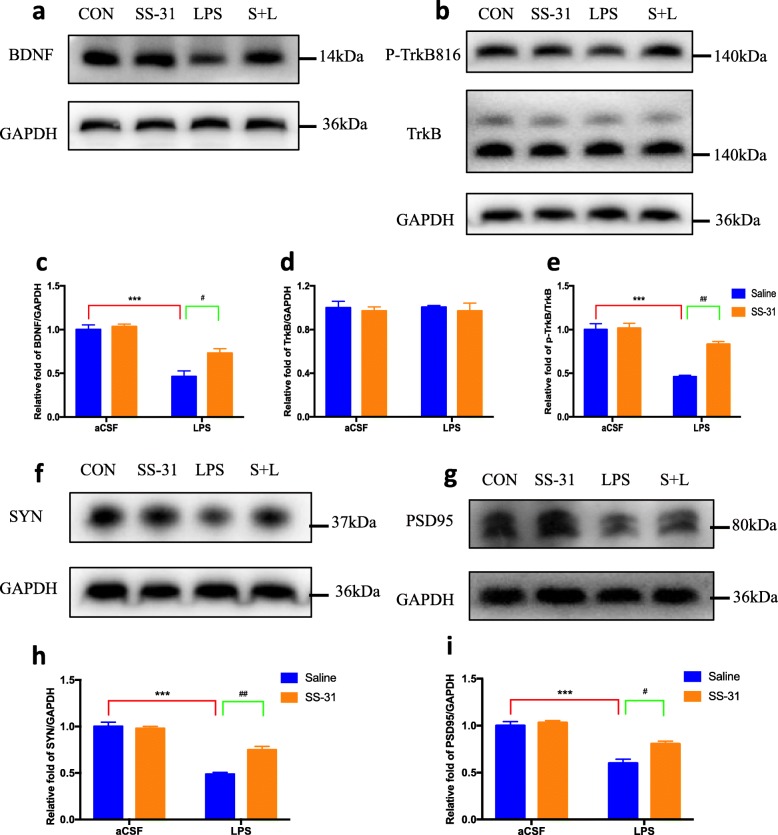
Effects of elamipretide (SS-31) on the BDNF pathway and synaptic plasticity in LPS-treated mice. **a**, **b** BDNF/TrkB pathway protein bands on the gel and their relative intensities. **c**–**e** The expression levels of BDNF and p-TrkB/TrkB proteins were normalized to that of GAPDH as an internal control. **f**, **g** Two kinds of synaptic-structural protein bands on the gel and their relative intensities. **h**, **i** The expression levels of SYN and PSD95 proteins were normalized to that of GAPDH as an internal control. Data are expressed as the mean ± SE (*n* = 6). ***P < 0.001 vs. CON group; #*P* < 0.05, ##*P* < 0.01 vs. LPS group. *SS*-*31* elamipretide, *aCSF* artificial cerebrospinal fluid, *LPS* lipopolysaccharide, *SE* standard error

To assess whether the balance of brain-derived neurotrophic factor (BDNF)/TrkB was affected by LPS, the expression of BDNF, TrkB, and p-TrkB proteins were measured using immunoblotting. As shown in Fig. 7a–e, the expression of BDNF protein was significantly decreased in the LPS-treated mice (*F*_LPS_ = 67.1, *P* < 0.001; Fig. 7c), whereas elamipretide treatment was able to restore the BDNF protein content to a level comparable to the CON group (*F*_SS-31_ = 8.66, *P* = 0.03; Fig. 7c). The total expression of TrkB protein showed no significant difference among the four groups (*F*_LPS_ < 0.001, *F*_SS-31_ = 0.393, *P* > 0.05; Fig. 7d). Further analysis of the p-TrkB/TrkB ratio demonstrated that the p-TrkB/TrkB ratio was decreased in the LPS group compared with the CON group (*F*_LPS_ = 58.4, *P* < 0.001, Fig. 7e). Elamipretide treatment significantly increased the p-TrkB/TrkB ratio in the hippocampus of the S + L group compared with that in the LPS group (*F*_SS-31_ = 16.8, *P* = 0.002; Fig. 7e).

SYN and PSD-95 are two important regulators and indicators for determining active synaptic plasticity. The results of immunoblotting showed that SYN (*F*_LPS_ = 128, *P* < 0.001; Fig. 7h) and PSD-95 (*F*_LPS_ = 78.6, *P* < 0.001; Fig. 7i) were downregulated in the LPS group, but elamipretide significantly attenuated the decrease of SYN levels (*F*_SS-31_ = 13.4, *P* = 0.002; Fig. 7h) and PSD-95 levels (*F*_SS-31_ = 11.5, *P* = 0.01; Fig. 7i).

These results suggest that elamipretide enhances the hippocampal BDNF pathway and maintains the formation of active synapses in mice with LPS-induced hippocampal impairment.

### Elamipretide prevented the reduction of dendritic spines on hippocampal neurons after LPS treatment

**Fig. 8:**
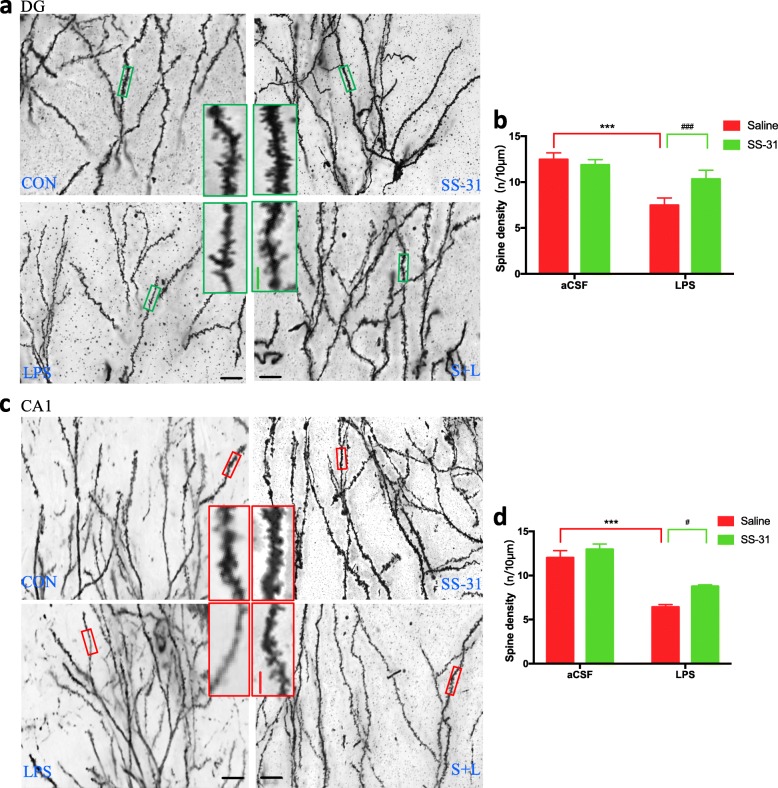
Alteration of dendritic spines on hippocampal neurons visualized with Golgi staining. **a**, **c** Golgi staining of dendritic spines in hippocampal dentate granule (DG) neurons and CA1 pyramidal neurons in indicated groups of mice. A magnified view of the green and red boxed areas in a and c is shown on the right side of the group (CON and the LPS groups). Left side of the group: elamipretide and S + L groups. **b**, **d** Number of dendritic spines per 10 μm on hippocampal dentate granule (DG) neurons and CA1 pyramidal neurons in indicated groups of mice. Scale bars in **a** and **c**, 20 μm; Inset boxes, 10 μm. Data are expressed as the mean ± SE (*n* = 6). ***P < 0.001 vs. CON group; #*P* < 0.05, ###*P* < 0.001 vs. LPS group. *SS*-*31* elamipretide, *aCSF* artificial cerebrospinal fluid, *LPS* lipopolysaccharide, *SE* standard error

It is well established that synaptic function is closely associated with the integrity of synaptic structure. Our results have shown that elamipretide can significantly attenuate the decreased expression of synaptic proteins, including SYN and PSD-95. We further explored whether elamipretide could change the morphology of dendritic spines, where synapses are located, when examined by Golgi staining. As shown in Fig. 8, the density of dendritic spines in hippocampal neurons, including granule neurons in the DG region area (green inset boxes in Fig. 8a) and pyramidal neurons in the CA1 region (red inset boxes in Fig. 8c), were significantly reduced in LPS-treated mice. In contrast, the reduced density of spines was attenuated by elamipretide treatment. When the number of dendritic spines per 10 μm was counted, we found that compared with the CON group, there were fewer spines on DG neuronal dendrites (*F*_LPS_ = 103, *P* < 0.001; Fig. 8b) and CA1 neuronal dendrites (*F*_LPS_ = 85.8, *P* < 0.001; Fig. 8d) in the LPS group, whereas elamipretide significantly attenuated the decrease of dendritic spines on DG neuronal dendrites (*F*_SS-31_ = 12.4, *P* = 0.002; Fig. 8b) and CA1 neuronal dendrites (*F*_SS-31_ = 9.83, *P* = 0.03; Fig. 8d). There was no overall alteration of the morphology of spines, and distinct types of spines (mushroom spines, stubby spines, and thin spines) could be seen in the four groups.

## Discussion

This study has shown that mice treated with LPS exhibited mitochondrial damage, oxidative stress response, inflammatory changes, neural cell apoptosis, and loss of dendritic spines in the hippocampus, leading to impaired hippocampus-related learning and memory performance. The behavioral tests showed that treatment with elamipretide significantly ameliorated LPS-induced hippocampus-dependent memory impairment. The protective effects of elamipretide on memory impairment in mice may be related to its mitochondria-targeted antioxidant properties, facilitating the modulation of BDNF signaling, and increasing the synaptic structural complexity in the mouse brain hippocampus.

Elamipretide is capable of successfully passing through the blood-brain barrier and targeting the inner mitochondrial membrane. It can be taken up extensively by cells and partitioned into mitochondria selectively. Intracellular concentrations of [^3^H]-elamipretide were found to be 6-fold higher than extracellular concentrations. Studies using isolated mitochondria revealed that [^3^H]-elamipretide is concentrated ~ 5000-fold in the mitochondrial pellet [13, 38]. Thus far, a wide array of neuroprotective effects of elamipretide has been reported in different studies. Previous studies have shown that elamipretide can protect neurons from degeneration in animal models and cell lines of Alzheimer’s disease (AD), Parkinson’s disease (PD), amyotrophic lateral sclerosis (ALS), multiple sclerosis (MS), and Friedreich ataxia (FA) that involve inflammatory and oxidative stress processes [16, 17, 23, 26, 31, 39]. In view of the evidence that oxidative stress and inflammatory responses play an important role in the mouse memory impairment model caused by LPS [4, 6, 30], our study focused on the effects of elamipretide in an LPS-induced memory impairment mouse model.

The MWM test and contextual fear conditioning were chosen as robust and reliable tests that are strongly correlated with hippocampal-dependent memory [32, 40–42]. We observed that LPS-induced memory impairments were prevented by elamipretide treatment (5 mg/kg ip). The results of the current study are consistent with a previous study that showed elamipretide pretreatment shortened the escape latency in training tests and increased the crossing-platform time and target quadrant time in a probe trial with the MWM test in developing rats exposed to isoflurane [25]. In a sepsis-associated encephalopathy mouse model, elamipretide attenuated the decrease of freezing time in the contextual fear conditioning [24]. Moreover, a significant decrease was observed in isoflurane mice compared with control mice in terms of the percentage of freezing time in the contextual fear conditioning, while mice pretreated with elamipretide showed a significant increase in the percentage of freezing time compared with the isoflurane group [43]. Overall, these findings suggest a potential application of elamipretide in patients with or at risk of memory impairment.

Since elamipretide is a mitochondria-targeted protectant, we first examined whether it would improve mitochondrial function in LPS-treated mice. Mitochondria from the hippocampus was isolated using the mitochondria isolation kit for tissue protocols. As important parameters of the mitochondrial function-related index, MMP and ATP levels were measured using an assay kit. Our results showed that elamipretide protected the hippocampus against LPS-induced mitochondrial dysfunction by maintaining MMP and ATP levels. It has been proposed that LPS induces mitochondrial dysfunction in experimental models [44, 45], and the present study revealed that the brain hippocampus is one of the most vulnerable areas. Although elamipretide may target mitochondria in other tissues, our results lend support to elamipretide protecting the hippocampus from mitochondrial dysfunction in LPS-treated mice. The results are in accordance with previous reports that elamipretide protects the integrity of mitochondria by maintaining the MMP level, and protects against the mitochondrial permeability transition pore (mPTP) opening in a sepsis-associated encephalopathy mouse model [24]. Additionally, a significant decrease was observed in isoflurane mice compared with the control mice in terms of the ATP content in the hippocampus, whereas mice pretreated with elamipretide showed a significant increase of ATP content compared with the isoflurane group [46]. Overall, treatment with elamipretide significantly improved mitochondrial function in LPS-treated mice.

Oxidative stress is defined as an imbalance between higher cellular levels of reactive oxygen species (ROS), e.g., superoxide and hydroxyl radicals, and cellular antioxidant defense [47, 48]. If ROS are not controlled by enzymatic and non-enzymatic antioxidants, they can cause oxidative injury, i.e., peroxidation of cell membrane phospholipids, proteins (receptors and enzymes), and DNA. The brain is extremely susceptible to oxidative damage induced by ROS because it generates very high levels of ROS due to its very high aerobic metabolism and blood perfusion, and its relatively poor enzymatic antioxidant defense [49]. Therefore, oxidative stress participates in neuronal injury and memory impairment. Consistently, elamipretide attenuated ROS accumulation induced by LPS exposure in the present study. Decreased activity of SOD and increased levels of MDA were found after LPS microinjection in the hippocampi of animals in the LPS group. Abnormal changes in the activity of SOD and levels of MDA were partially reversed by elamipretide, suggesting that the neuroprotective effects of elamipretide might be related to its antioxidant effects. Considerable evidence implicates neuroinflammation in the pathophysiology of progressive neurodegenerative disorders [50, 51] and a link between increased cytokine formation and neurodegeneration has been demonstrated [52]. Several studies have demonstrated that mitochondrial reactive oxygen species (mtROS) are essential for priming the activation of NF-κB and the NOD-like receptor family, pyrin domain containing 3 (NLRP3) inflammasome [53, 54], which activate caspase 1 and result in processing and secretion of the pro-inflammatory cytokines. NF-κB is also reported to be an essential signal that promotes the expression of NLRP3 and substrates TNF-α and IL-6 [53]. Previous studies have shown that LPS triggers microglia activation and consequently induces pro-inflammatory cytokine secretion within 6 h via the NF-κB pathway in the mouse hippocampus, and that the nuclear signal of the transcription factor is strongly reduced or blocked by anti-inflammatory compounds [55, 56]. Thus, it is rational that scavenging mtROS by elamipretide could suppress the increases of TNF-α and IL-6 in LPS-treated mice. Elamipretide possesses the ability to block NF-κB activation and exert a neuroprotective effect, suggesting that the neuroprotective effects of elamipretide might be related to its anti-neuroinflammation effects. In fact, oxidative stress and neuroinflammation exist simultaneously, and the interaction between oxygen free radicals and inflammatory factors aggravates cognitive deficiency [57, 58].

It is well known that LPS causes cognitive lesions and that this process involves apoptosis. As we have reported previously, LPS-induced memory impairment is associated with decreased Bcl-2 expression and increased neural cell apoptosis [6]. In this study, mitochondrial dysfunction was found after exposure to LPS, but treatment with elamipretide significantly improved mitochondrial integrity and function by maintaining MMP and ATP levels in the hippocampus of mice. Overproduction of mtROS inhibits mitochondrial electron transport, which may result in mitochondrial membrane depolarization and the initiation of apoptosis by releasing Cyt C [59]. The present study provided evidence that the mitochondrion-targeted antioxidant elamipretide significantly inhibited apoptosis and prevented memory impairment in LPS-treated mice. Thus, protection against mitochondrial dysfunction by removing mtROS will prevent the activation of the intrinsic mitochondria-dependent apoptotic pathway and further reduce hippocampus-dependent memory defects in LPS-treated mice. These results indicate that mitochondria play an important role in oxidative stress and neuroinflammation induced cognitive impairment.

BDNF and its signaling pathways are firmly implicated in neuronal differentiation and survival. Numerous pieces of evidence indicate that BDNF regulates both the early and late phases of long-term potentiation in the hippocampus [60]. Its regulation of synaptic plasticity may underlie hippocampus-dependent learning and memory. BDNF exerts its neuronal protective functions through binding to the TrkB receptor. BDNF/TrkB has been shown to regulate the phosphorylation, trafficking, and expression of *N*-methyl-d-aspartate (NMDA) receptor subunits. The actions of BDNF on NMDA receptors in the hippocampus have direct implications in its ability to facilitate Ca^2+^ influx [43]. Studies have previously reported its role in learning, memory, and neurogenesis [61–64]. BDNF signaling through postsynaptic TrkB receptors is essential for the insertion of AMPARs (AMPA-type receptors) for postsynaptic density and the development of mature synapses [61]. Overall, BDNF/TrkB signaling is critical for synaptic function and plasticity. The synapse-associated proteins, especially presynaptic SYN and postsynaptic PSD-95, promote synaptic plasticity [37, 65–67]. Aberrant dendrites and spines in the hippocampus are related to neurodegenerative disorders [36]. In this study, the levels of presynaptic SYN and postsynaptic PSD-95 decreased in the hippocampus of mice, accompanied by the downregulated BDNF/TrkB signaling after the mice were treated with LPS. Elamipretide treatment modulated and rectified BDNF/TrkB signaling and increased the levels of presynaptic SYN and postsynaptic PSD-95 in mice with hippocampal impairment. Golgi staining showed that LPS decreased dendritic spine density, but elamipretide prevented the reduction of dendritic spines on hippocampal neurons after LPS treatment in the hippocampal CA1 and DG regions. Consistently, previous studies have also reported that abnormal hippocampal neuronal plasticity was found following LPS-induced neuroinflammation [15, 68]. This LPS-induced decrease in synaptic protein levels may contribute to impairment of synaptic plasticity and the learning and memory decline observed in behavioral tests, suggesting impairments of synaptic plasticity may be responsible for LPS-induced memory deficits. Meaningfully, in our study, treatment with elamipretide prevented the LPS-induced reduction of SYN and PSD-95 might through modulating the BDNF/TrkB signaling pathway. Elamipretide protected against the reduction of dendritic spines on hippocampal neurons might facilitate the protective effects of LPS-induced learning and memory impairment.

Thus, elamipretide treatment consolidated the hippocampal BDNF/TrkB signaling pathway by selectively reversing the protein expression or phosphorylation, and protected against mitochondrial dysfunction, and it specifically rescued the structure and function of the synapse against hippocampus-dependent learning and memory impairment.

## Conclusion

In conclusion, our in vivo studies indicate that LPS-induced memory impairment can be attenuated by the mitochondrion-targeted antioxidant elamipretide. Its protective mechanism may be not only related to its mitochondria-targeted antioxidant activity but also the modulation of BDNF/TrkB signaling, including increasing synaptic structural complexity. Therefore, elamipretide may have a therapeutic potential in preventing the damage from oxidative stress and neuroinflammation that contributes to memory impairment, which makes mitochondria a potential target for treatment strategies for neuroinflammation-related cognitive impairment. Further investigation of the neuroprotective activity of elamipretide in the mammalian system is warranted, given the prospect that this medication will be used for prophylactic, as well as adjuvant therapy for neurodegenerative diseases resulting from oxidative and inflammatory damage.

## Data Availability

The datasets during and/or analyzed during the current study are available from the corresponding author on reasonable request.

## Abbreviations

aCSF: Artificial cerebrospinal fluid
AD: Alzheimer’s disease
AKI: Acute kidney injury
ALS: Amyotrophic lateral sclerosis
ANOVA: Analysis of variance
ATP: Adenosine triphosphate
BDNF: Brain-derived neurotrophic factor
BSA: Bovine serum albumin
CA1: Cornu ammonis 1
CKD: Chronic kidney disease
CS: Conditioned stimulus
DAPI: 4′,6-Diamidino-2-phenylindole
DCFH-DA: Dichloro-dihydrofluorescein diacetate
DG: Dentate gyrus
dUTP: Deoxyuridine triphosphate
ELISA: Enzyme-linked immunosorbent assay
FA: Friedreich ataxia
FCT: Fear conditioning test
FITC: Fluorescein isothiocyanate
HRP: Horseradish peroxidase
IL-6: Interleukin-6
LPS: Lipopolysaccharide
MDA: Malondialdehyde
MMP: Mitochondrial membrane potential
mPTP: Mitochondrial permeability transition pore
MS: Multiple sclerosis
mtROS: Mitochondrial reactive oxygen species
MWM: Morris water maze
NBT: Nitro blue tetrazolium
NLRP3: NOD-like receptor family, pyrin domain containing 3
OFT: Open field test
PD: Parkinson’s disease
PMSF: Phenylmethylsulfonyl fluoride
PND: Perioperative neurocognitive disorders
PSD-95: Postsynaptic density protein 95
PVDF: Polyvinylidene difluoride
RIPA: Radioimmunoprecipitation assay
ROS: Reactive oxygen species
SDS–PAGE: Sodium dodecyl sulphate–polyacrylamide gels
SE: Standard error
SOD: Superoxide dismutase
SS-31: Szeto-Schiller-31/elamipretide/MTP-131/Bendavia/RX-31/D-Arg-Dmt-Lys-Phe-NH2
SYN: Synaptophysin
TBA: Thiobarbituric acid
TLR4: Toll-like receptor 4
TNF-α: Tumor necrosis factor α
TrkB: Tropomyosin receptor kinase B
TUNEL: Terminal transferase biotinylated-dUTP nick end labeling
US: Unconditioned stimulus
WB: Western blotting
